# Parasite-induced hormonal manipulation associated with severe male reproductive costs in a wild mammal

**DOI:** 10.1098/rspb.2026.2229

**Published:** 2026-11-11

**Authors:** Medhavi Verma, Lorin Hutchings, Michael Baumgarten, Benjamin C. Trumble, Sharmi Sen, Alemayehu Lemma, Firehewot Ayele, Abebaw Azanaw, Fanuel Kebede, Fekede Regassa, Belayneh Abebe, Amy Lu, Jacinta C. Beehner, Thore J. Bergman, Noah Snyder-Mackler, India A. Schneider-Crease

**Affiliations:** ^1^ School of Human Evolution and Social Change, Arizona State University, Tempe, AZ, USA; ^2^ Center for Evolution and Medicine, Arizona State University, Tempe, AZ, USA; ^3^ Institute of Human Origins, Arizona State University, Tempe, AZ, USA; ^4^ School of Life Sciences, Arizona State University, Tempe, AZ, USA; ^5^ School of Technology for Public Health, Arizona State University, Tempe, AZ, USA; ^6^ Department of Anthropology, Northwestern University, Evanston, IL, USA; ^7^ Department of Anthropology, University of Michigan, Ann Arbor, MI, USA; ^8^ Department of Psychology, University of Michigan, Ann Arbor, MI, USA; ^9^ Department of Ecology and Evolutionary Biology, University of Michigan, Ann Arbor, MI, USA; ^10^ Addis Ababa University College of Veterinary Medicine and Agriculture, Debre Zeit, Ethiopia; ^11^ Simien Mountains Park Office, Ethiopian Wildlife Conservation Authority, Debark, Ethiopia; ^12^ Ethiopian Wildlife Conservation Authority, Addis Ababa, Ethiopia; ^13^ Department of Anthropology, Stony Brook University, Stony Brook, NY, USA

**Keywords:** parasite manipulation, life history trade-offs, fitness costs, steroid hormones, reproductive success, primates, *Taenia*

## Abstract

The most spectacular examples of parasite manipulation are those that induce conspicuous and lethal behavioural changes to accelerate host death and facilitate parasite transmission. Far less understood are subtler forms of manipulation that enhance parasite fitness within living hosts, inflicting substantial reproductive costs without immediately compromising survival. In the laboratory, certain parasites have been shown to manipulate the host endocrine system to increase their own growth at immense reproductive cost to hosts; however, this relationship has been challenging to detect in nature because its effects can mimic natural variation and require long-term, integrative data in wild host–parasite systems. Here, we leverage nearly two decades of data linking infection, physiology, behaviour and fitness in wild gelada monkeys infected with the larval tapeworm *Taenia serialis*. We show that males with evidence of recent infection had 65% higher plasma oestradiol and a 237% increase in the risk of losing reproductive access, translating to approximately 1.5 fewer offspring. These same males maintain or increase, rather than decrease, levels of plasma testosterone and faecal androgen metabolites, respectively, suggesting that compensatory mechanisms may be operating in this evolutionary arms race between parasites and hosts.

## 1. Introduction

Parasite manipulation is most famously illustrated by striking, lethal changes in host behaviour that facilitate transmission from one host to another. The ecological and behavioural mechanisms underlying this strategy, which often exploits predator–prey relationships and requires the death of the prey host for transmission, are relatively well-characterized across taxa [1–4]. Far less understood, however, are subtler forms of manipulation that enhance parasite fitness within *living* hosts; these strategies can carry substantial costs that fall short of increasing mortality and can thus be more challenging to detect, particularly in the wild.

Experimental work has shown that some parasites can manipulate the endocrine environment of their hosts for their own benefit and at a reproductive cost to their hosts. For example, the asexually budding larvae of the *Taenia crassiceps* tapeworm preferentially proliferate in oestradiol-rich environments, die when subjected to testosterone [5] and actively promote the conversion of testosterone to oestradiol in male mouse hosts by upregulating host-derived cytochrome P-450 aromatase [6,7]. This manipulation confers fitness benefits for the parasite by increasing reproductive output in an initially hostile endocrinological environment while inhibiting male mouse testosterone and reproduction [8–10]. By contrast, *T. crassiceps* has a much more muted effect on the hormonal profiles of female mice that already provide sufficiently oestradiol-rich environments [11,12]. Despite its evolutionary significance, evidence for this type of strategy is currently limited to laboratory settings [9,11], in part because detecting these effects in wild systems requires extensive and integrative data on infections, sex hormones and reproductive success over lifespans.

Building on experimental work with *T. crassiceps*, we take the study of parasite manipulation to a natural system: wild primates infected with the asexually budding larval stage of *T. serialis*, a tapeworm related to *T. crassiceps* [13]. Geladas (*Theropithecus gelada*) ingest eggs shed by carnivores infected with the gut-living adult tapeworm, each of which can yield multiple larvae in gelada flesh through asexual budding. Infection-associated morbidity and mortality in geladas facilitate predation or scavenging by carnivores, allowing larvae to mature into gut-living adults in the carnivore host (supplementary material, figure S1) [14,15]. Larval budding in *T. serialis*, like in *T. crassiceps*, increases the number of potential tapeworm adults transmitted to the next host. If *T. serialis* modifies the gelada endocrine environment as *T. crassiceps* does in the mouse environment, infection could inflict fitness costs for males beyond direct effects on morbidity and mortality by impeding reproductive access.

Gelada male reproductive success is tied to the ability to monopolize reproductive access to a group of females through physical competitions with other males [16–19]. Males disperse from their natal groups (‘reproductive units’ comprising related adult females and immature offspring) into all-male, non-reproductive ‘bachelor’ groups [20–22]. These bachelor groups are peripheral to the relatively stable ‘band’ of associating reproductive units and engage with units primarily to challenge incumbent ‘leader males’ who have near-exclusive reproductive access during their tenure [23–25]. During challenges, bachelors and leaders engage in energetically costly vigorous physical and vocal displays and contact aggression [16,22,24,26]. Following a successful challenge (‘takeover’), the ascendant bachelor assumes the leader position and mates with the unit's adult females (ranging between one and 12 in this population [27]). The reproductive urgency posed by the time-limited leadership tenure is reflected in high rates of infanticide [27,28] and post-takeover foetal loss that may allow females to recoup anticipated infanticide costs [29]. Overthrown males can remain in the unit as ‘followers’ with few reproductive opportunities, rejoin bachelor groups or succumb to takeover-associated injuries [16,23]. Testosterone probably plays a critical role in competition; faecal androgen metabolites are higher among leaders than bachelors throughout the year—particularly those with more receptive females [22]—but become highest in bachelors during the takeover period [16]. Because reproductive access in gelada males is associated with specific endocrine patterns, parasite-associated endocrinological manipulation could severely impact male reproductive success.

We leverage two decades of behavioural, endocrine and *T. serialis* infection data on wild geladas in the Simien Mountains National Park, Ethiopia, to test the central hypothesis that *T. serialis* manipulates its male hosts to establish an oestradiol-rich endocrine environment conducive to larval survival and growth, inflicting reproductive costs on its hosts that rely on androgen-associated competitive phenotypes. We predict that, in gelada males, infection is associated with (i) higher plasma oestradiol, (ii) lower plasma testosterone, (iii) lower faecal androgen metabolites and (iv) higher risk of losing leadership tenure. We expect that the endocrine effects will be observable in males but not females because females already offer an oestradiol-rich environment conducive to larval survival and growth. Our findings provide rare evidence for endocrine manipulation of male hosts in a natural setting and demonstrate severe infection-associated reproductive costs for males. Altogether, these results provide empirical evidence that parasite manipulation may shape variation in male reproductive success in a wild mammal.

## 2. Results

### (a) Active *T. serialis* infection associated with higher male plasma oestradiol

Infection with *T. serialis* (i.e. positive plasma antigen assay) was associated with a 65% increase in male plasma oestradiol (exponentiated estimate = 1.65, *p* = 0.001, adj. *R*^2^ = 0.33; figure 1, supplementary material, table S3) but not with any change in testosterone (exponentiated estimate = 1.14, *p* = 0.765, cond. *R*^2^ = 0.35, marg. *R*^2^ = 0.16; figure 1, supplementary material, table S4), controlling for the fixed effects of body weight, male life history status (i.e. bachelor, follower and leader) and age. This included 28 unaffiliated ‘bachelor/natal’ males and eight subordinate ‘follower’ males; leaders are not immobilized and thus not sampled to avoid disrupting reproductive units. Of 36 male plasma samples analysed for oestradiol (mean = 68.8 pg ml^–1^, range = 21.9–171.1 pg ml^–1^) and testosterone (mean = 3.3 ng ml^–1^, range = 0.3–15.6 ng ml^–1^; cross-reactivity in supplementary material, table S1, coefficients of variation in supplementary material, table S2), 12 (33.3%) were *Taenia* antigen-positive, of which four had protuberant cysts characteristic of late-stage infection. In parallel analyses of female plasma oestradiol (mean = 111.0 pg ml^–1^, range = 40.4–417.74 pg ml^–1^) and testosterone (mean = 0.43 ng ml^–1^, range = 0.19–1.36 ng ml^–1^), infection was not associated with either hormone (30 cycling adult females, 23% antigen-positive, five visible cysts; supplementary material, figure S2, tables S5–S6).

**Figure 1.**
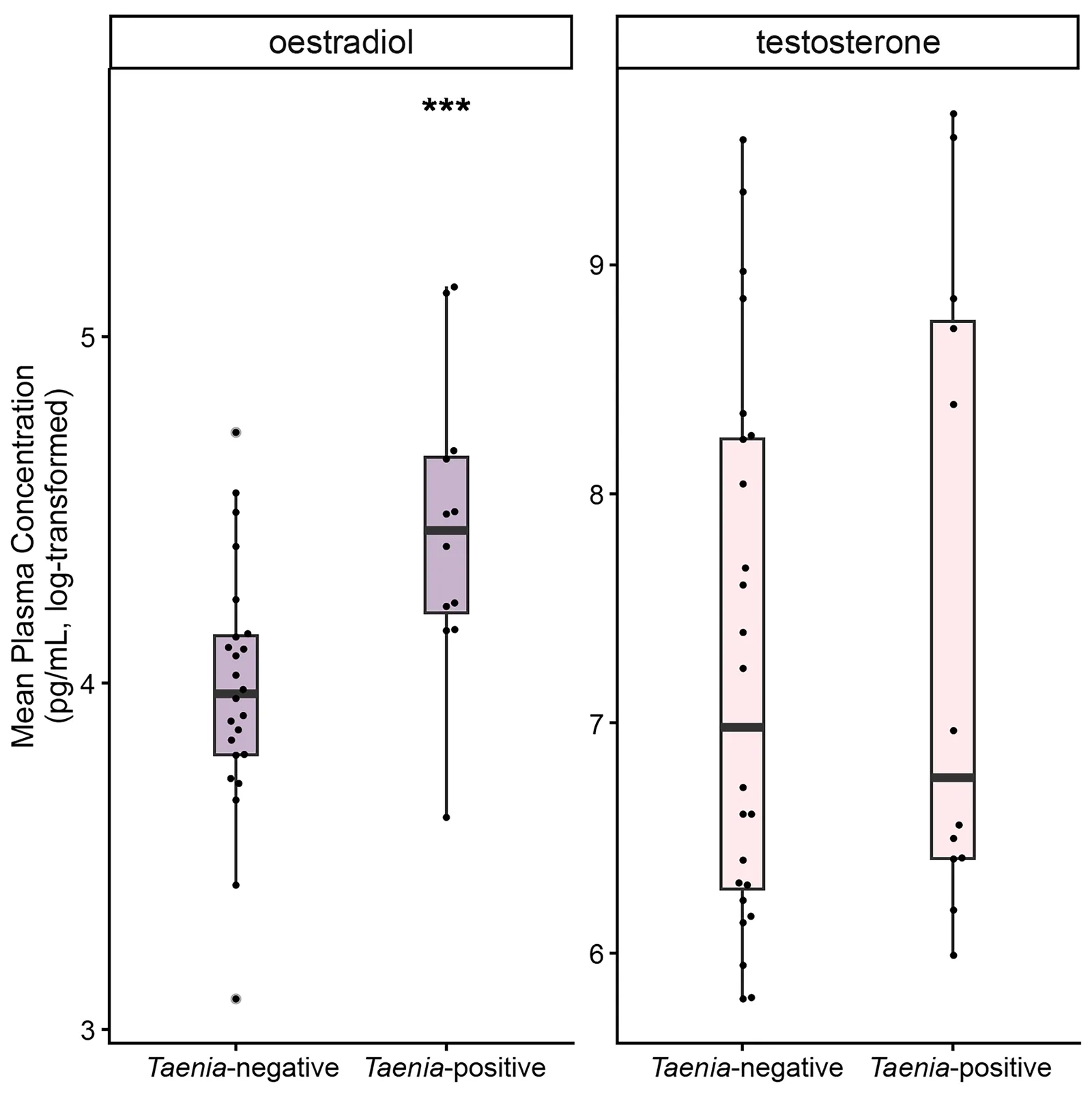
Larval *T. serialis* infection is associated with higher oestradiol (estimate = 0.50, *p* = 0.001), but not testosterone (estimate = 0.13, *p* = 0.765), in males. Each point represents a single plasma sample (*n* = 36, of which 24 were *Taenia*-negative and 12 were *Taenia*-positive). The boxes represent the interquartile range, the horizontal lines represent the median values and the whiskers represent the ranges of values within 1.5 times the interquartile range.

### (b) Recent evidence of *T. serialis* infection associated with higher male faecal androgen metabolites

Recent evidence of infection (i.e. visible cyst or positive *Taenia* antigen sample within the prior year) was associated with 27.8% higher faecal androgen metabolite concentration in leaders (exponentiated estimate = 1.28, *p* = 0.004; cond. *R*^2^ = 0.38, marg. *R*^2^ = 0.21; figure 2, supplementary material, table S8) after accounting for social status and season in linear mixed models with log-transformation. There was a near-significant interaction between infection and bachelor status (exponentiated estimate = 0.71, *p* = 0.087; supplementary material, table S8). We then performed pairwise comparisons of marginal means within each male status to estimate differences in faecal androgen metabolites between males with and without recent evidence of infection. Leaders with recent evidence of infection had significantly higher faecal androgen metabolites than those without (estimated difference = 0.251, *p* = 0.005), but no significant differences were detected within followers (*p* = 0.420) or bachelors (*p* = 0.636; supplementary material, table S9). Analyses on more restrictive datasets with cyst observation or urine or plasma collection and faecal sample collection within one-month (exponentiated estimate = 1.42, *p* = 0.039), three-month (exponentiated estimate = 1.46, *p* = 0.013) and six-month (exponentiated estimate = 1.25, *p* = 0.046) windows also showed significant effects in the same direction but had larger standard errors and wider confidence intervals. Of 399 faecal androgen metabolite samples (mean = 754.2 ng g^–1^, range = 102 ng g^–1^–2681.7 ng g^–1^; see cross-reactivity in supplementary material, table S7; published in [21]) matched with the most recent urine or plasma antigen test within a year or cyst presence for each male, 143 (35.8%) from 22 males had recent evidence of infection (84 from leaders, nine from bachelors and 50 from followers).

**Figure 2.**
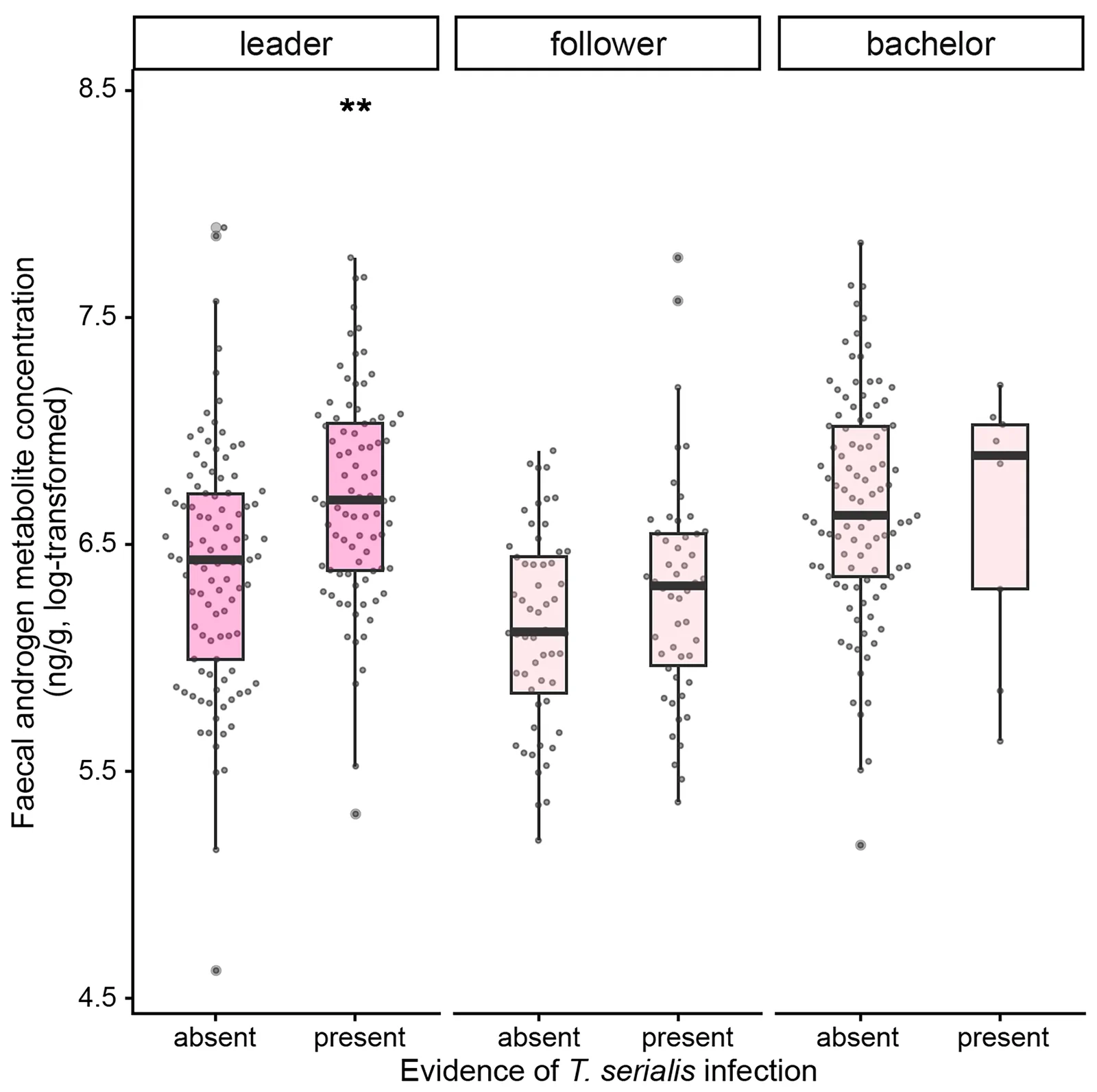
Infection with larval *T. serialis* is associated with higher concentrations of faecal androgen metabolites in leader males (estimate = 0.25, *p* < 0.01). Each point represents a single faecal sample (*n* = 399, of which 143 had recent evidence of infection and 256 did not). The boxes represent the interquartile range, and the whiskers represent the range of values within 1.5 times the interquartile range.

### (c) Recent evidence of *T. serialis* infection associated with increased risk of male tenure loss and fewer offspring

Recent evidence of infection increased the instantaneous hazard of tenure loss by approximately 3.37-fold (a 237% increase, hazard ratio (HR): 3.37, *p *< 0.001, 95% CI = 1.97–5.78; figure 3a, supplementary material, table S10) after accounting for the effect of age (HR: 1.36, *p* < 0.001) and number of follower males (HR: 1.39, *p* < 0.001), with the model stratified by binned group size. A male-level percentile bootstrap with 5000 replicates produced a similar confidence interval for the infection hazard ratio (HR = 3.37, 95% bootstrap CI = 1.65–6.37). The Cox proportional hazards model included 3067 monthly intervals (rows) across 103 tenures from 96 males; 87 tenures ended in tenure loss and 16 were right-censored. Recent evidence of infection was recorded for 175 intervals (including 11 tenure losses), while the remaining 2892 intervals were classified as having no recent evidence of infection and included 76 tenure losses. Because infection was designated by month and could vary over the course of a tenure (e.g. a male can have multiple months without evidence of infection followed by multiple months with evidence of infection), the same male or tenure could contribute to the 175 ‘recent evidence of infection’ intervals and the 2892 ‘no recent evidence of infection’ intervals. Mean tenure length across the entire dataset was approximately 2.5 years (29.8 months) and median tenure length was approximately 1.8 years.

**Figure 3.**
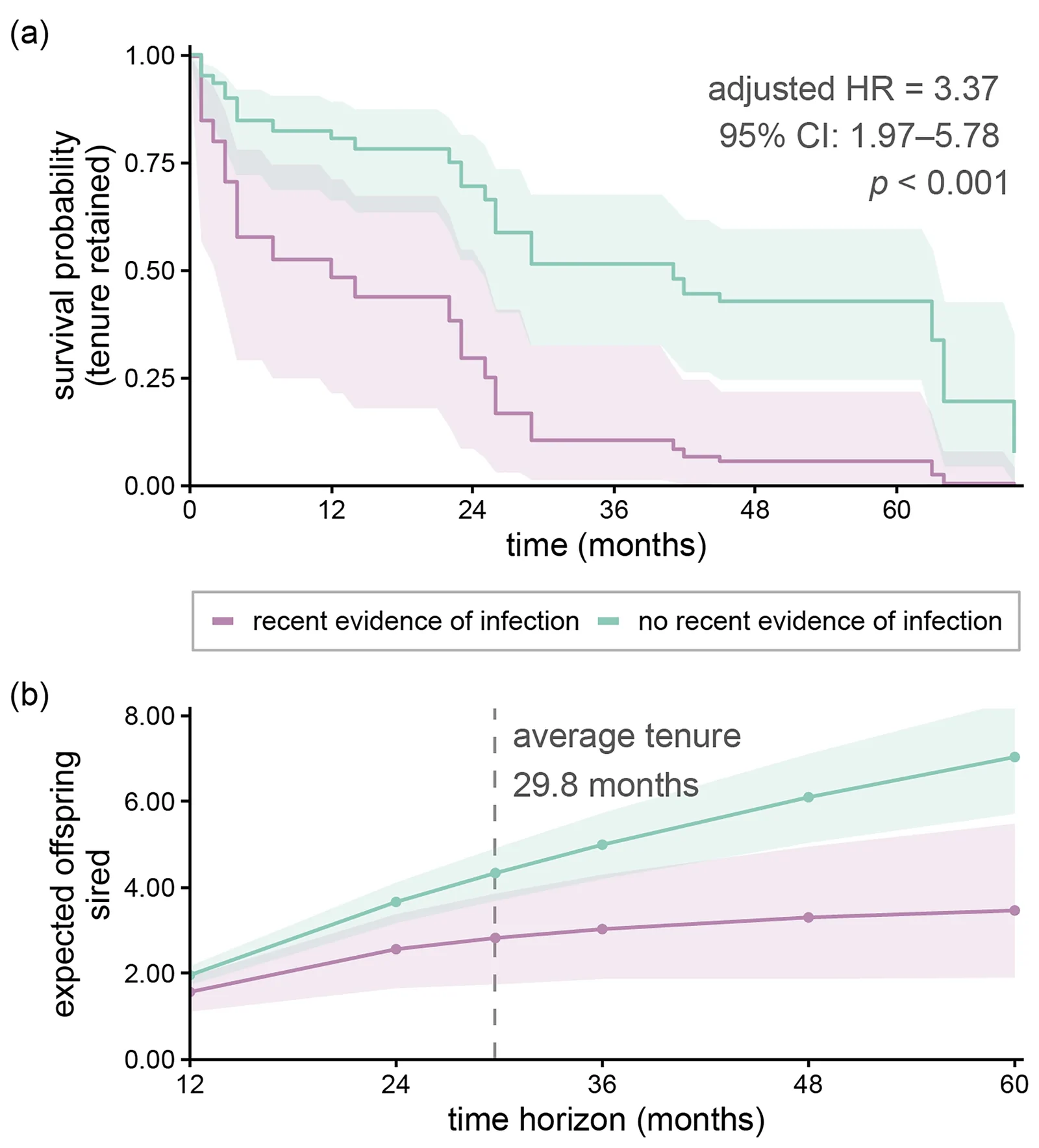
Recent evidence of *T. serialis* infection is associated with reproductive costs for male geladas. (a) Adjusted conditional survival curves show the predicted probability of retaining leader tenure for males with recent evidence of infection (purple, reflecting a positive antigen test within the preceding year or a current cyst) and no recent evidence of infection (green). Curves are predicted from the primary Cox proportional hazards model showing a 3.37-fold (237%) increase in risk of tenure loss with recent evidence of infection, based on a representative male with the median starting age (approx. 10.5 years) and number of follower males (0) in a medium-sized group. Age increases across the prediction period, while other covariates remain fixed. (b) Expected offspring sired over 12–60-month time horizons, calculated by converting restricted mean survival time estimates from the same model using average reproductive parameters observed in this population. Shading represents 95% confidence intervals; intervals in (a) are model-based, while intervals in (b) were estimated from 1000 male-level bootstrap samples.

Males with recent evidence of infection were estimated to have 1.51 fewer offspring by the mean tenure end (95% CI: 0.53–2.56) and 3.57 fewer offspring by 60 months (95% CI: 1.50–5.19) based on restricted mean survival time (RMST) analysis. RMST estimated that males with recent evidence of infection accumulated 2.12 fewer months of retained tenure than males without recent evidence of infection within the first 12 months of tenure (figure 3b, supplementary material, table S11), increasing to 8.08 months lost by the mean tenure length and 19.61 months lost by 60 months. Expected offspring numbers for each male tenure were calculated separately under the two infection categories as RMST multiplied by the number of females present at the start of the tenure divided by the average interbirth interval. Offspring lost was the difference between the estimates of the two infection categories.

## 3. Discussion

Active larval *T. serialis* infection or recent evidence of infection was associated with a 65% increase in circulating oestradiol in follower and bachelor males, an approximately 3.37-fold (237%) increase in risk of reproductive tenure loss for leader males, and approximately 1.5 potential offspring lost by the average tenure duration. Contrary to the low levels of testosterone expected from parasite-induced aromatization to oestradiol, recent evidence of infection was associated with higher faecal androgen metabolites in leaders and no detectable change in bachelor and follower males. Similarly, active infection was not associated with any change in plasma testosterone in bachelors and followers (leaders were not sampled for plasma). This suggests that infected leader males may be under pressure to compensate for testosterone loss by upregulating testosterone production, which may compound the costs of infection itself and ultimately contribute to the severe impact on reproductive tenure. The impact of *T. serialis* on the reproductive success of its male hosts, probably associated with its manipulative strategy, presents an example in the wild of a widely assumed and theoretically expected relationship with limited empirical evidence in mammals. Altogether, the evidence presented here for costly endocrine manipulation demonstrates that parasites with complex life cycles can have multidimensional effects on mammalian host fitness that go beyond the primary impact of increased mortality for enhanced transmission.

While infection and illness can broadly reduce sex hormone levels by catalysing trade-offs between immunity and reproduction via inhibition of the hypothalamic–pituitary–gonadal (HPG) axis [30,31], our results show infection-associated increases in oestradiol that are consistent with expectations for parasite-induced endocrine manipulation. Following initial sickness-associated increases in oestradiol as the body aromatizes costly and potentially immunosuppressive levels of testosterone, both hormones should then decline as resources are diverted [31,32]. By contrast, the observed 1.6-fold higher oestradiol levels in *T. serialis*-infected males than in uninfected males persisted even during late-stage infections represented by characteristic cysts. This suggests that, like the larvae of its asexually budding congeneric in the laboratory, *T. serialis* larvae in the wild manipulate host endocrine profiles to produce an oestrogenic environment that increases asexual proliferation.

Oestradiol-associated increases in larval cell proliferation in the taeniid family may result from the upregulation of the AP-1 transcription factor via oestrogen-receptor-like proteins on the parasite surface or through a MAP Kinase/ERK pathway via a putative G-protein-coupled receptor [5,33–35]. Oestrogen receptor signalling may also assist in cytoskeleton assembly and movement, which are integral for parasite homeostasis and survival [36]. High levels of oestradiol can create a permissive environment for larval growth by dampening the activity of the inflammatory immune response through the induction of a Th2-type immune phenotype [34,37]. In the androgenic male host, taeniid-induced aromatization of testosterone to oestradiol through stimulation of aromatase cytochrome P-450—potentially through an IL-6-mediated immunoendocrine pathway [38]—would thus directly promote parasite fitness. The lack of infection-associated hormonal changes in females indicates that the parasites do not need to adopt this strategy in naturally oestrogen-rich environments, although we were unable to control for oestrogenic noise associated with menstrual stages.

Diverging from the lower testosterone levels seen in male mice infected with *T. crassiceps*—and from expectations based on sickness-associated HPG inhibition—*T. serialis* was not associated with lower testosterone in geladas. Rather, infection was associated with maintained (for bachelors and followers, measured in plasma) or increased (for leaders, measured in faecal androgen metabolites) testosterone. These unexpected androgen patterns could be attributed to parasite-associated disruption in HPG feedback loops (figure S3). If *T. serialis* infection depletes testosterone through aromatization and uses the resulting oestradiol for proliferation, the lack of negative feedback (signalling sufficient testosterone) would trigger the secretion of gonadotropin-releasing hormone (GnRH), production of luteinizing hormone (LH) and follicle-stimulating hormone (FSH) and renewed testosterone synthesis [39]. That testosterone would again be aromatized and the oestradiol depleted by *T. serialis*, continuing the cycle, which could produce the observed lack of testosterone depletion in the plasma and the increased androgen metabolites in the faeces of males with recent evidence of infection. Because lab mice are subjected to high-intensity experimental infections, this type of HPG axis-mediated testosterone compensation may not be sufficient to produce the pattern we observe in geladas.

The infection-associated increase in faecal androgen metabolites for leaders—but not for followers and bachelors—may reflect a status-specific pressure to continuously invest in a high-testosterone phenotype. Non-leader males may be able to more readily reallocate resources away from sex hormone production to immune function, although the lack of testosterone depletion in infected bachelors and followers suggests that they may maintain baseline investment rather than entirely redirecting resources. Males with higher baseline testosterone may also be intrinsically better at sustaining androgen-mediated behaviours or compensating for aromatization; higher-testosterone males would thus be overrepresented in the leader dataset, which could contribute to the observed higher faecal androgen metabolites in leaders. Alternatively, higher faecal androgens in leaders with recent evidence of infection could potentially reflect increased oestrogens because the measured metabolite (epiandrosterone) is derived from the dehydroepiandrosterone pathway that gives rise to both sex hormones [39]. However, because our plasma assay—which has a greater specificity than the faecal assay—showed increased infection-associated oestradiol in non-leaders, we would expect to observe increased faecal androgen metabolites in those males if the assay was detecting increased oestrogens. Because we do not observe that effect, higher faecal androgens most probably reflect higher testosterone and thus point to the compensatory strategy in leaders.

Parasite manipulation has been described in all major parasitic taxa, affecting hosts across the animal and plant kingdoms [1,2,4]. Most research has focused on behavioural manipulation that increases the vulnerability of hosts to predation or increases mortality in other ways [40]. Through facilitating the consumption of one host by another (i.e. predation and scavenging), this strategy transmits larvae from the prey host to the predator host, where larvae can develop into their next developmental stage. While phenotypic changes in hosts can occur during non-manipulative infections, those that result from manipulation alone should diverge from sickness-based expectations and should carry discernible fitness benefits for the parasite [3]. Based on these criteria, our results suggest manipulation by *T. serialis*: infection is associated with a phenotypic change in sex hormones that diverges from the expected global decline in sex hormones associated with infection, and this change is consistent with conditions that would benefit the parasite by increasing survival and proliferation via asexual budding that increases the number of adult parasites in the next generation. These effects are not expected for parasites without an asexually proliferative stage; for example, infections with the non-asexually budding *T. solium* larvae are associated with decreases in both hormones in men and male boars [41,42], aligning with the expected sickness phenotype [30,43] rather than host manipulation. Our results thus provide evidence from the natural world for endocrine manipulation within a single host.

Although parasitism has long been considered an important component of life history evolution and population dynamics through impacts on host fitness [44], our findings join a sparse literature of parasite-associated male reproductive costs in wild mammals [45–47]. The severe hazard of tenure loss associated with recent evidence of *T. serialis* infections in geladas translates directly to loss of reproductive opportunities and lower male fertility, which are reflected in the predicted loss of approximately 8 months of tenure and approximately 1.5 offspring by the average end of tenure in this population (30 months). Because these calculations assumed no follower males, which both increase the risk of tenure loss and may each claim a share of offspring sired, these estimates probably underestimate the reproductive costs for males in groups with followers. Most males struggle to become leader again after an initial defeat: only seven of the 96 leader males in our dataset secured a second leader position (with second tenures 65% shorter than the first), meaning little chance of recouping fitness losses after losing tenure. This effect may arise in part from an energetic crisis precipitated by manipulation of the endocrine phenotype. The continued investment by leader males in a high-testosterone phenotype (e.g. vocal and physical displays) may compound the immunological costs of *T. serialis* larval growth in somatic and visceral tissue. Even with continued investment in testosterone production, infected males may have limited energetic reserves with which to defend their leadership, increasing the likelihood of being targeted or losing challenges.

The reproductive costs of *T. serialis* for males may be mediated by the timing of infection. The highest costs are probably borne by males infected early in their lives, who may be unable to levy successful challenges to incumbent leader males and be entirely precluded from reproduction. Indeed, we have never observed a bachelor with a cyst become a leader. Within leaders, timing should mitigate the selective pressure of infection. Some leaders showed evidence of infection within a year of their tenure start and had the greatest number of expected offspring lost, while others showed evidence of infection only after years of leadership and siring multiple offspring. This wide variation may lessen the strength of selective pressure exerted by *T. serialis* on male geladas and slow the evolution of counterstrategies.

The strength of these analyses lies in the integration of long-term data on behaviour, plasma hormones and faecal androgen metabolites, but each dataset carries limitations inherent to research in natural systems. While the plasma hormone assays are more specific and sensitive than the faecal androgen metabolite assay, darting wild primates presents challenges that non-invasive faecal collection does not. We refrained from darting leader males to minimize our impact on the social group and sampled individuals only once; thus, our plasma dataset is cross-sectional and comprises only bachelors and followers, excluding males expected to occupy the highest end of the testosterone range. On the other hand, faecal samples can be collected non-invasively from all males [16,20,21], but the assay detects an androgen metabolite rather than a hormone itself and thus its interpretation is less straightforward. Integrating the two leverages their complementary strengths but does not eliminate their respective limitations. In the same vein, we cannot control for the exact oestrous stage for females, which could contribute to variation in female hormonal profiles and mask an effect of infection.

Our classification of *T. serialis* infection carries similar trade-offs. Visual observation of the characteristic cysts associated with infection that have been recorded since the inception of the research project permits an extended look-back time but excludes early infection stages and internally developing infections. In addition, negative antigen results may miss active infections because cyst calcification or intermittent antigen release can reduce detectability [48]. Conversely, positive results in males without visible cysts may reflect infections that are subsequently eliminated rather than becoming established [49], although these dynamics need further investigation across taeniids and within the gelada–*T. serialis* system [50]. Integrating these infection metrics over time periods of interest allows us to statistically evaluate the relationship between *T. serialis,* hormones and tenure loss, but adds additional complexity to their interpretation.

Despite these limitations, our findings provide, to our knowledge, the first evidence from a wild mammal consistent with parasite-driven endocrine manipulation. Together, these findings illuminate a cryptic dimension of parasite–host relationships with the potential to shape evolutionary trajectories of each. Our findings also reveal extraordinary reproductive costs for male hosts, offering rare empirical support for a long-standing and widely held theoretical prediction that has been challenging to demonstrate in wild mammals. Finally, because asexually budding larval tapeworms also pose a threat to human health [51,52], these findings further illuminate the biological processes underlying severe and often neglected human diseases.

## 4. Materials and methods

### (a) Subjects and study site

All data were collected from a population of habituated geladas in the Simien Mountains National Park, Ethiopia (SMNP; 13.183°N, 38.0667°E), where the Simien Mountains Gelada Research Project (SMGRP) has studied a population of approximately 300 geladas in approximately 22 reproductive units (3240 m a.s.l.) since 2006. Researchers collect near-daily behavioural, demographic, reproductive and health data on individually recognizable individuals. Ages are known or estimated based on secondary sexual characteristics [53]. Male status and status changes are assigned based on continuous behavioural observation: bachelor males live in all-male groups and follower males are behaviourally submissive in mixed-sex groups where leader males have near-exclusive mating access [16]. All research and procedures were conducted with permission from the Ethiopian Wildlife Conservation Authority and approved by the Institutional Animal Care and Use Committee at the University of Washington (protocol 4416-01) and Arizona State University (20-1754R). All analyses were performed in R v. 4.4.1 [54].

### (b) Plasma sampling

We collected plasma from geladas during temporary immobilizations between 2017 and 2020. Geladas were immobilized via a blowpipe (Telinject USA, Inc.) with ketamine (7.5 mg kg^–1^) and medetomidine (0.04 mg kg^–1^). Anaesthesia was reversed with atipamezole (0.2 mg kg^–1^) within an hour of sedation, and individuals were monitored until they returned to their social groups. Darting was generally constrained to morning hours (approx. 7 am–12 pm) to allow sufficient recovery time before dark. We collected approximately 4–5 ml of whole blood from sedated animals via femoral venipuncture into anticoagulant-treated EDTA tubes that were inverted 4–5 times. Samples were immediately placed on ice in a cooler and centrifuged within 3–5 hours of collection (1000 × g, room temperature, 10 minutes) at the field laboratory. 1 ml of plasma was then separated into 2 ml cryovials, which were flash-frozen in liquid nitrogen for transport. Samples were stored in liquid nitrogen for up to three weeks, shipped to Arizona State University in liquid nitrogen vapour (approx. 3–4-day transport time) and stored at –80℃ until analysis. Samples were stored from collection (2017, 2019 and 2020) until analysis in 2024.

### (c) *T. serialis* infection

Infection with *T. serialis* can be identified via observation of characteristic protuberant cysts or via a monoclonal antibody-based *Taenia* antigen-detection enzyme-linked immunosorbent assay (ELISA) using plasma or urine. Cysts are visually distinctive, never resolve and can be tracked visually over time, while the ELISA captures earlier infection stages, advanced stages developing internally and potentially early-stage infections that can be eliminated prior to cyst establishment [55]. Urine samples were collected non-invasively from all group males between 2013 and 2020, while plasma samples were collected from a limited subset of follower and bachelor males between 2017 and 2020 (as detailed in §4b). Because our analyses integrate multiple types of data over variable time periods, we use operational infection categories in statistical analyses that include long-term data. In our retrospective analysis of faecal androgen metabolites, the Cox proportional hazards analysis for tenure loss, and the estimation of offspring lost based on restricted mean survival time, we used ‘recent evidence of infection’ to capture males with cysts and males whose last urine or plasma sample was *Taenia*-positive in the preceding year. We used ‘no recent evidence of infection’ to capture males without cysts who had a *Taenia*-negative urine or plasma sample for the faecal androgen metabolites analysis or males who either had a *Taenia*-negative urine or plasma sample or had no recent urine or plasma sample in that same window for the Cox proportional hazards and restricted mean survival time analyses (see §4f for further explanation).

Longitudinal records of visible *T. serialis* cysts span 2006–2023 and include males across reproductive statuses. Urine was collected non-invasively from the ground using Whatman grade 4 filter paper, dried for shipment and re-eluted prior to assay [55], with data available for males of all statuses–including repeated sampling of the same males over time—from 2013–2020. Plasma was collected and stored as described in §4b from bachelor and follower males from 2017–2020. Assays followed established protocols [55–58], with sample type-specific preparation steps. Urine was reconstituted from filter paper in blocking buffer (PBS-Tween + 1% NBCS, as described in [55]), while plasma was diluted 1 : 9 in blocking buffer. Plates were run in the Division of Parasitic Diseases and Malaria, Centers for Disease Control and Prevention (CDC), in Atlanta, Georgia (supplementary material S3). Samples were designated as positive if their optical density was equal to or greater than that of the positive controls.

### (d) Plasma hormone analysis

We used DRG enzyme immunoassay kits to assess testosterone (Kit EIA-1559 [59]) and oestradiol (Kit EIA-2693 [60]) concentrations in plasma samples according to the manufacturer's instructions on the first freeze–thaw cycle (supplementary material S1 and S2; testosterone cross-reactivity in supplementary material, table S1). Standards, in-house controls and samples were run in duplicate. Spike recovery was assessed prior to analysis and fell within acceptable ranges for both hormones (testosterone: 95–111% recovery (mean = 102%); oestradiol: 78–101% (mean = 88%)). Control values and coefficients of variation were within recommended ranges (supplementary material, table S2). One sample did not react in either of the two assays and was excluded prior to dataset assembly. We also excluded samples from females with high oestradiol and progesterone who were likely to be pregnant and from those likely to be lactating (i.e. with dependent offspring), cross-checked against birth and weaning records. Our final dataset included 36 males (28 bachelors, 8 followers) and 30 cycling adult females. No plasma samples are available for leader males because we avoid darting them to minimize our impact on the social group.

### (e) Long-term faecal data collection

This dataset included 399 faecal samples collected across the years 2008–2020 from 39 adult males (10.2 mean samples per male) that had been sampled for *T. serialis* in plasma or urine, or who had presented with characteristic cysts. This included 185 samples from leaders, 108 from bachelors and 106 from followers. Faecal samples were collected, stored and assayed for faecal androgen metabolites using epiandrosterone enzyme immunoassays, as published in [21] (see supplementary material, table S7 for cross-reactivities from [61]). Because samples are collected non-invasively, they allow for repeated sampling of all males over time; however, this assay cannot directly measure oestradiol and is subject to unknown variation owing to individual metabolism and excretion [61].

### (f) Reproductive tenure

The final reproductive dataset contained 3067 observations from 96 males representing 103 unique tenures from 2006–2023, with 87 observed tenure losses. Tenures averaged 2.5 years (914 days, range: 30–3195 days). We excluded individuals with tenures shorter than 30 days (*n* = 6) and assigned unique tenure IDs to account for seven males that had two separate tenures. Sixteen individuals were right-censored in this dataset. Thirty-one rows from three males contained missing values for the number of follower males present and the number of females in the group that month. We imputed these values using predictive mean matching in the ‘mice’ package [62] to complete the dataset and retain these rows. Dataset totals exclude three males without known or estimated dates of birth.

A month was classified as having recent evidence of infection if the male had a visible cyst during that month or if his most recent antigen test within the preceding year was positive (*n* = 175 months, 19 males; six with cysts, 14 with positive antigen tests, one with both a cyst and a positive test). We use a 12-month window to capture recent active infection. 214 months had a negative antigen test in that window (*n* = 17 males), and 2678 months had no test or visible cyst within that window. Because only two tenure-loss events occurred during months with a confirmed negative test in the preceding year, negative and unknown months could not be evaluated separately. Thus, all months with a recent negative result were combined with months with no test and no cyst to form the ‘no recent evidence of infection’ category and allow for comparison with the months with recent evidence of infection. As a sensitivity analysis, we refitted the Cox model using a more restrictive six-month window.

### (g) Statistical analyses

#### (i) Infection and plasma hormones: linear and linear mixed models

We used linear and linear mixed effects models to assess the impact of *T. serialis* infection on plasma oestradiol and testosterone, respectively [63,64]. For males, we modelled each log-transformed averaged hormone concentration as a function *of T. serialis* infection (0/1), social status (bachelor/natal or follower), body mass (kg, continuous) and age (categorical) as common predictors of hormones [16,20,21,65]. We combined bachelor and natal males from study groups who were in the process of transitioning to bachelor groups at the time. We analysed data from females similarly, with *T. serialis* infection (0/1) and body mass (kg, continuous) as fixed effects. Sampling year was included as a random effect in both testosterone models but not in either oestradiol model, where it explained no variance and led to a singular model fit. Model diagnostics indicated that all assumptions were met [66,67]. One outlier in the female oestradiol analysis was removed, which did not modify results.

#### (ii) Infection and faecal hormone metabolites: linear mixed model

We modelled log-transformed faecal androgen metabolite concentration (ng g^–1^) using a linear mixed-effects model [63,64]. After conducting model selection on a set of plausible models using AICc [68], our final model included *T. serialis* infection status (0/1) based on the most recent urine or plasma sample within the previous year or prior observation of a cyst, social status (leader, bachelor or follower) and season (hot–dry, cold–wet and cold–dry) [69] to account for other variation in faecal androgen metabolites in this population (see supplementary material, table S13 for model comparisons). The interaction between infection and status was also included to account for contrasting relationships between infection and faecal androgen metabolites across leaders, bachelors and followers. Individual ID and sampling year were included as random effects. We then performed pairwise comparisons of marginal means using the Kenward–Roger method for degrees of freedom to estimate the differences in faecal androgen metabolites between males with and without recent evidence of infection within each male status (leaders, followers and bachelors) [70]. We used a 1-year window because faecal and urine sampling occurred at different paces, and this window allowed for inclusion of the largest number of males; experimentation with more restrictive temporal constraints reduced power but showed the same relationship. We performed the same analysis on more restrictive datasets (i.e, samples within one-, three- and six-month windows) to confirm the direction and significance of effects on more constrained temporal scales. Model diagnostics indicated that all assumptions were met [67]. All variance inflation factors (‘vif’ function, ‘car’ package) were below 1.5, indicating low multicollinearity among predictors [66].

#### (iii) Survival analysis

We modelled whether recent evidence of *T. serialis* infection was associated with the hazard of leader tenure loss using a Cox proportional hazards model fitted with the ‘coxph’ function in the *survival* package [71]. All dates were aligned to monthly intervals; events occurring before the 15th day of a month were assigned to the first day of that month and events occurring on or after the 15th were assigned to the first day of the following month. We then structured each tenure as monthly start–stop intervals beginning at 0–1. The event variable remained 0 until the interval in which tenure loss occurred, which was coded as 1. Tenures that had not ended by the end of observation were right-censored.

The primary model included recent infection evidence, age in years at each month and the number of follower males as time-varying covariates. We stratified the model by female group size [27]: small (1–4 females), medium (5–7 females) or large (8–12 females). Rows from the same tenure were grouped together by unique tenure identification, and robust variance estimates clustered by male accounted for repeated monthly observations and multiple tenures from the same male. Males without known or estimated dates of birth (*n* = 3) were excluded prior to analysis. We used Efron's method for tied event times.

We assessed proportional hazards using scaled Schoenfeld residuals, finding no evidence of violations for any predictor (global test: *p* = 0.18) [72] and detected no departure from linearity for covariates. We calculated 95% confidence intervals and *p*-values using cluster-robust standard errors clustered by male. As an additional sensitivity analysis, we calculated a 95% percentile bootstrap confidence interval for the hazard ratio with 5000 male-level replicates with replacement. Each bootstrap sample retained all rows and tenures per male, and the primary Cox model was refitted for every replicate.

#### (iv) Restricted mean time lost

We estimated the number of offspring lost associated with recent evidence of *T. serialis* infection based on restricted mean survival time (RMST) [73]. For each observed tenure start, we used the predicted tenure retention under the two scenarios in the primary Cox model (recent evidence of infection and no recent evidence of infection within the preceding year). The predicted curves used the observed age, allowing it to progress over time, assumed the group-size category at tenure start remained constant, and assumed no followers. We calculated RMST at mean observed tenure duration (29.8 months) and at 12, 24, 36, 48 and 60 months. Estimates were averaged across tenures, with each male given equal weight. We converted RMST to offspring lost by multiplying tenure months retained by the number of females at tenure onset, dividing by the population average interbirth of approximately 30.84 months [28], and calculating the difference between the conditions. We estimated 95% confidence intervals using 1000 male-level percentile bootstrap samples with group size fixed at the starting value for each tenure, refitting the primary Cox model and repeating the RMST and offspring-lost calculations.

## Supplementary Material



## Data Availability

Data and code to reproduce these analyses are available on Zenodo [74]. Supplementary material is available online [75].
