# Supplementary material for: Parasite-induced hormonal manipulation associated with severe male reproductive costs in a wild mammal
Source: Proc Biol Sci. 2026 Nov 11;293(2082):20262229. doi: 10.1098/rspb.2026.2229 (PMC13751119; doi:10.1098/rspb.2026.2229)
Supplement: Supplementary file 1 [file rspb.2026.2229.zip › rspb.2026.2229/rspb20262229_si_001.docx]

Supplementary Materials for

**Parasite-induced hormonal manipulation associated with severe male reproductive costs in a wild mammal**

**The file includes:**

Supplementary Materials and Methods

Figs. S1 to S3

Tables S1 to S12

**Materials and Methods**

Plasma estradiol and testosterone concentrations were measured using commercial competitive enzyme immunoassays (DRG Estradiol EIA-2693 and Testosterone EIA-1559) following the manufacturer’s protocols (52). Samples, standards, and controls were incubated with enzyme conjugate at room temperature, washed, and developed with substrate. Absorbance was measured at 450 nm with a reference wavelength of 620-630 nm. Estradiol samples were diluted in blocking buffer, and spike-recovery controls were included to validate assay performance.

Recent evidence of *Taenia* infection was measured using a monoclonal antibody antigen-capture ELISA. Plates were coated with W8 capture antibody and incubated with plasma diluted 1:10. Bound antigen was detected with biotinylated W5 antibody followed by streptavidin-HRP. Plates were developed with TMB for 5 min, stopped, and read at 650 nm. Positive and negative serum controls were included on each plate.

Detailed protocols for all assays are provided below.

Estradiol Enzyme ImmunoAssay (DRG EIA-2693)

[**Kit**](https://store.drg-international.com/shop/estradiol-elisa.asp): Estradiol serum/plasma solid-phase, competitive enzyme-linked immunosorbent assay (ELISA) *(52)*.

1) Prepare samples/bring all reagents to room temp
2) Add 25 µL of standard, control, and sample to wells
   a) 100µL ETA blocking buffer in tubes 1:2-1:64
   b) 100µL serum to 100µL block
   c) Spike recovery: put 100µL of neat serum in 3 tubes labeled “low”, “med”, “high”. Add standards as follows:
      i) Low = 10µL s0 standard. Vortex.
      ii) Med = 10µL s6 standard. Vortex.
      iii) High = 10µL s7 standard. Vortex.
3) Add 100 µL of Enzyme conjugate to wells
   a) Plate shaker (10 seconds)
4) Incubate 90 min at room temperature
5) Rinse 3x with 400µL wash solution
   a) Shake out
6) Add 100 µL of substrate solution to wells
7) Incubate 30 min at room temperature
8) Add 50 µL of stop solution to wells
9) Read plates at 450nm and approx. 620nm-630nm within 10 minutes of previous step

Testosterone Enzyme ImmunoAssay (DRG EIA-1559)

[**Kit**](https://store.drg-international.com/shop/testosterone-elisa.asp): Testosterone serum/plasma solid-phase, competitive enzyme-linked immunosorbent assay (ELISA) *(52)*.

1) Prepare samples/bring all reagents to room temp
2) Add 25 µL of standard and sample to wells
3) Add 200 µL Enzyme Conjugate to wells
   a) Plate shaker (10 seconds)
4) Incubate 60 min at room temperature
5) Rinse 3x with 400µL wash solution
   a) Shake out
6) Add 200µL of substrate solution to wells
7) Incubate 15 min at room temperature
8) Add 100µL stop solution
9) Read plates at 450nm and approx. 620nm-630nm within 10 minutes of previous step

Protocol for *Taenia* assay in plasma

Materials:

1) Chemicals/reagents
   a) W5 monoclonal detecting antibody-biotinylated
   b) W8 monoclonal capturing antibody
   c) NaH_2_PO_4_.H_2_O
   d) Na_2_HPO_4_.7H_2_O
   e) NaCl
   f) Tween20 (Calbiochem cat. #655205)
   g) Deionized water (dH_2_O)
   h) Dry milk powder (Nestle Carnation Instant Nonfat Dry Milk Fortified with Vitamins A + D)
   i) SureBlue^TM^ TMB [3,3'5,5' tetramethylbenzidine] Microwell Peroxidase Substrate (1-Component) (Kirkegaard & Perry, Gaithersburg, MD, cat No. 52-00-00)
   j) TRIS (C_4_H_11_NO_3_)
   k) Blue Stop
   l) KCl
   m) EDTA, Na salt
   n) HCl
   o) Serum controls:
      i) Negative: Normal human serum
      ii) Positive: Positive control serum
2) Supplies
   a) Immulon 2 HB plates
   b) 2 x 50 ml graduated cylinder
   c) 3 x 50 ml beakers
   d) Stir bars
   e) 1 x 15 ml centrifuge tube
   f) 6 reusable troughs
   g) Pipette tips 1-200 μl
   h) Kay-Dry paper towels and Kim-Wipe tissues (Kimberly-Clark).
   i) Vinyl examination gloves
3) Instrumentation
   a) vMax microplate reader with computer equipped with SOFTmax for Windows software for reader control and data analysis, and printer (Molecular Devices Corp)
   b) Vortex-Genie vortex mixer (Fisher Scientific)
   c) Magnetic plate stirrer (Fisher Scientific)
   d) 8-  or 12-channel variable volume 20-200 μl pipette
   e) 10 μl pipette
   f) Bellco mini-orbital shaker (Bellco Glass)

Steps:

1) Thaw samples from -80 at 37C and keep on ice
2) Sensitize the Nunc Medisorp plate with 100 µL of 1 µg/mL of W8 in Tris-HCl, pH 8 + 2 mM EDTA + 1 M KCl for 2 hours at RT, covered, shaking
3) Make serum dilution (1:10) in PBS-Tw-milk
   a) Shake to mix
4) Wash the plate with 4 x 300 µL of PBS/0.3% Tween
5) Add 100 ul of sample dilution
6) Shake, covered, for 30 min
7) Prepare W5-Biotin conjugate: 1:1000 biotin-W5 in PBS-Tw-milk
   a) Vortex and mix end over end
8) Take out TMB and Blue-Stop from 4C
9) Prepare Streptavadin-HRP conjugate (1:1000) in PBS/Tween/milk
10) Wash plate as in Step 4
11) Add 100 µL of W5-biotinylated diluted 1:1000 in PBS/Tween/milk
12) Incubate for 30 min RT, shaking
13) Wash the plate as in Step 4
14) Add 100 µL of Streptavidin-HRP 1:1000 in PBS/Tween/milk
15) Incubate for 30 min RT, shaking
16) Wash as in Step 4
17) Add 100 µL of TMB
18) Incubate for 5 min, RT, shaking, uncovered
19) Add 100 µL of Blue Stop
20) Read at 650 nm

**Fig. S1.** Visible cyst characteristic of *T. serialis* (left) on a wild female gelada in the Simien Mountains National Park, Ethiopia, and a depiction of the hypothesized full life cycle (right). The definitive host of *T. serialis* in this system remains unknown.

**Fig. S2. Infection was not associated with hormone levels in adult cycling females.** No significant effect of *Taenia* infection was detected on oestradiol (estimate = 0.13, p = 0.662) or testosterone (estimate = 0.22, p = 0.308) within cycling females.

**Fig. S3**. Male geladas infected with *T. serialis* do not show a decline in testosterone despite increased parasite-induced aromatization, potentially owing to continued synthesis by the hypothalamic–pituitary–gonadal (HPG) axis. Putative mechanism: testosterone is depleted by the parasite via increased aromatization to oestradiol. The lack of negative feedback from sex hormones to the hypothalamus and pituitary gland allows the HPG axis to continue synthesizing testosterone, thus explaining the increase in oestradiol and maintained or raised testosterone levels.

**Table S1.** Cross-reactivity for the DRG Testosterone ELISA (EIA1559) used to assess plasma testosterone as reported by the manufacturer.

| **Analyte** | **Cross-reactivity** |
| --- | --- |
| Testosterone | 100 |
| 5α-Dihydrotestosterone | 0.8 |
| Androstenedione | 0.9 |
| 11β-Hydroxyestosterone | 3.3 |
| 17α-Methyltestosterone | 0.1 |
| 19-Nortestosterone | 3.3 |
| Epitestosterone | < 0.1 |
| Oestradiol | < 0.1 |
| Progesterone | < 0.1 |
| Cortisol | < 0.1 |
| Oestrone | < 0.1 |
| Danazol | < 0.1 |

**Table S2.** Coefficients of variation (CVs) for plasma steroid hormone ELISAs.

| **Hormone** | **Between-plate CV** | | | **Within-plate CV** |
| --- | --- | --- | --- | --- |
|  | **Low in-house control** | **Medium in-house control** | **High in-house control** |  |
| Testosterone | 6.02% (0.4 ng/mL) | 5.12% (0.4 ng/mL) | 2.996% (3.4 ng/mL) | 3.71% |
| Oestradiol | 16.85% (82.8 pg/mL) | 8.79% (145.4 pg/mL) | 8.54% (226.1 pg/mL) | 8.49% |

**Table S3.** Linear model results for analysis of *T. serialis* plasma infection and log-transformed oestradiol (pg/mL) in male geladas (adj. R^2^ = 0.33). Bachelor and natal males from the study group who were in the process of transitioning to bachelor groups during sampling were analyzed in one group and compared to follower males.

| **term** | **estimate** | **std. error** | **t value** | **p. value** |
| --- | --- | --- | --- | --- |
| (Intercept) | 4.04 | 0.54 | 7.45 | <0.001 |
| Taenia-positive | 0.50 | 0.14 | 3.64 | 0.001*** |
| Body mass (kg) | 0.01 | 0.02 | 0.42 | 0.683 |
| Age (categorical, subadult vs adult) | 0.21 | 0.21 | 1.00 | 0.327 |
| Bachelor/natal (categorical, vs followers) | -0.42 | 0.16 | -2.64 | 0.013* |

**Table S4.** Linear mixed model results for analysis of *T. serialis* plasma infection and log-transformed testosterone (ng/mL) male geladas (cond. R^2^ = 0.35, marg. R^2^ = 0.16). Bachelor and subadult natal males from the study group who were in the process of transitioning to bachelor groups during sampling were analyzed in one group and compared to follower males.

| **term** | **estimate** | **std. error** | **df** | **t value** | **p. value** |
| --- | --- | --- | --- | --- | --- |
| (Intercept) | -1.38 | 1.70 | 30.99 | -0.81 | 0.424 |
| Taenia-positive | 0.13 | 0.43 | 30.22 | 0.30 | 0.765 |
| Body mass (kg) | 0.11 | 0.08 | 29.93 | 1.43 | 0.163 |
| Age (categorical, subadult vs adult) | -0.26 | 0.63 | 29.40 | -0.41 | 0.685 |
| Bachelor/natal (categorical, vs followers) | 0.07 | 0.48 | 29.63 | 0.15 | 0.885 |

**Table S5.** Linear model results for analysis of *T. serialis* plasma infection and log-transformed oestradiol (pg/mL) in female geladas (adj. R^2^ = -0.06).

| **term** | **estimate** | **std. error** | **t value** | **p. value** |
| --- | --- | --- | --- | --- |
| (Intercept) | 4.73 | 0.86 | 5.46 | < 0.001 |
| Taenia-positive | 0.13 | 0.29 | 0.44 | 0.662 |
| Body mass (kg) | -0.02 | 0.07 | -0.33 | 0.747 |

**Table S6.** Linear mixed model results for analysis of *T. serialis* plasma infection and log-transformed testosterone (ng/mL) in female geladas (cond. R^2^ = 0.16, marg. R^2^ = 0.04).

| **term** | **estimate** | **std. error** | **df** | **t value** | **p. value** |
| --- | --- | --- | --- | --- | --- |
| (Intercept) | -0.79 | 0.65 | 25.40 | -1.21 | 0.237 |
| Taenia-positive | 0.22 | 0.21 | 26.94 | 1.04 | 0.308 |
| Body mass (kg) | -0.02 | 0.05 | 26.77 | -0.37 | 0.713 |

**Table S7.** Cross-reactivity for the Epiandrosterone Enzyme Immunoassay used for fecal androgen metabolite assessment reported in [59].

| **Analyte** | **Cross-reactivity** |
| --- | --- |
| Epiandrosterone | 100 |
| Androstandione | 800 |
| Androsterone | 40 |
| Androstendione | 15.6 |
| Norandrostendione | 10.4 |
| Dehydroepiandrosterone | 7.4 |
| 5-Androsten-3,17-dione | 7 |
| 5β-Androstan-3,17-dione | 2.3 |
| 5α-Androstan-3α,17β-diol | 1.4 |
| 5α-Androstan-3β,17β-diol | 1.3 |
| 5α-Dihydrotestosterone | 1.3 |
| 5α-Androstan-17α-ol-3-one | 1.1 |
| 5α-Androstan-3α,17α-diol | 0.2 |
| 5α-Androstan-3β,17α-diol | 0.1 |
| Etiocholanolone | < 0.1 |
| Oestrone | < 0.1 |

**Table S8.** Linear mixed model results for analysis of recent evidence of *T. serialis* infection and fecal androgen metabolite concentration (ng/g) (cond. R^2^ = 0.38, marg. R^2^ = 0.21).

| **term** | **estimate** | **std. error** | **df** | **t value** | **p. value** |
| --- | --- | --- | --- | --- | --- |
| (Intercept) | 6.42 | 0.09 | 30.41 | 73.38 | <0.001 |
| Taenia-positive | 0.25 | 0.09 | 242.84 | 2.93 | 0.004** |
| Follower | -0.37 | 0.11 | 79.40 | -3.35 | 0.001** |
| Bachelor | 0.22 | 0.10 | 48.15 | 2.19 | 0.033* |
| Cold-wet season | 0.06 | 0.06 | 368.79 | 1.06 | 0.292 |
| Hot-dry season | 0.26 | 0.06 | 247.24 | 4.63 | <0.001*** |
| Taenia-positive: Follower | -0.14 | 0.15 | 113.25 | -0.91 | 0.364 |
| Taenia-positive: Bachelor | -0.34 | 0.20 | 217.57 | -1.72 | 0.087 |

**Table S9.** Pairwise comparisons of estimated marginal means within male statuses for the faecal androgen metabolites model [68]. Positive and negative indicate recent evidence of infection and no recent evidence of infection, respectively. Degrees of freedom were calculated using the Kenward-Roger method.

|  | **Leader** | | **Follower** | | **Bachelor** | |
| --- | --- | --- | --- | --- | --- | --- |
| **Infection status** | **-** | **+** | **-** | **+** | **-** | **+** |
| **Marginal means** | 6.53 | 6.78 | 6.15 | 6.27 | 6.76 | 6.67 |
| **SE** | 0.09 | 0.1 | 0.11 | 0.11 | 0.1 | 0.17 |
| **df** | 21 | 30.1 | 41.7 | 43.9 | 29.4 | 216.4 |
| **CI** | 6.35 - 6.72 | 6.59 - 6.98 | 5.94 - 6.37 | 6.05 - 6.48 | 6.55 - 6.96 | 6.33 - 7.01 |
|  | | | | | | |
| **Estimated difference** | 0.25 | | 0.11 | | -0.09 | |
| **SE** | 0.09 | | 0.14 | | 0.18 | |
| **df** | 236.9 | | 69.5 | | 187.9 | |
| **t ratio** | -2.862 | | -0.81 | | 0.48 | |
| **p value** | 0.005 | | 0.420 | | 0.636 | |

**Table S10.** Cox proportional hazards models of tenure loss associated with recent evidence of *T. serialis* infection. The primary model used a 12-month detection window and combined unknown and negative months as having no recent evidence of infection for males without visible cysts. The sensitivity analysis used the same structure with a 6-month detection window.

| **Model** | **Predictor** | **Estimate** | **Hazard ratio** | **95% CI** | **p-value** | **Concordance** |
| --- | --- | --- | --- | --- | --- | --- |
| primary model (12-month window, negative and unknown combined) | recent evidence of infection | 1.22 | 3.37 | 1.97-5.78 | <0.001 | 0.658 (SE = 0.04) |
|  | age | 0.31 | 1.36 | 1.16-1.61 | <0.001 |  |
|  | number of follower males | 0.33 | 1.39 | 1.22-1.58 | <0.001 |  |
| sensitivity model (6-month window, negative and unknown combined) | recent evidence of infection | 1.12 | 3.05 | 1.63–5.70 | <0.001 | 0.658 (SE = 0.04) |
|  | age | 0.30 | 1.36 | 1.15-1.60 | <0.001 |  |
|  | number of follower males | 0.32 | 1.37 | 1.21-1.57 | <0.001 |  |
| Confidence intervals and p-values were calculated using cluster-robust standard errors to account for repeated observations | | | | | | |

**Table S11.** Restricted mean survival times (RMST) based on population-averaged curves for males with and without recent evidence of infection. The difference between RMST for positive and non-positive equals the number of months lost associated with infection. Number of offspring lost are calculated by multiplying the number of lost months by reproductive parameters (# females per group, interbirth interval) observed in this population. Confidence intervals are provided in parentheses.

| **Measure** | **12 months** | **24 months** | **29.8 months (mean tenure length)** | **36 months** | **48 months** | **60 months** |
| --- | --- | --- | --- | --- | --- | --- |
| RMST: no recent evidence detected, months | 10.86  (10.04 - 11.45) | 20.41  (18.34 - 22.01) | 24.27  (21.42 - 26.56) | 28.03  (24.41 - 31.05) | 34.42  (29.36 - 38.83) | 39.76  (33.48 - 45.66) |
| RMST: recent evidence detected, months | 8.74  (6.19 - 10.44) | 14.56  (9.38 - 18.76) | 16.18  (9.90 - 21.59) | 17.45  (10.36 - 24.15) | 19.18  (10.78 - 28.40) | 20.15  (11.01 - 31.24) |
| Months lost | 2.12  (0.56 - 4.19) | 5.85  (1.91 - 10.29) | 8.08  (2.91 - 13.44) | 10.58  (3.88 - 16.58) | 15.25  (6.26 - 22.43) | 19.61  (8.37 - 28.02) |
| Expected offspring: no recent evidence detected | 1.96  (1.74 - 2.18) | 3.66  (3.18 - 4.12) | 4.34  (3.70 - 4.92) | 5.00  (4.20 - 5.73) | 6.10  (5.04 - 7.11) | 7.04  (5.72 - 8.35) |
| Expected offspring: recent evidence detected | 1.57  (1.11 - 1.93) | 2.57  (1.66 - 3.38) | 2.83  (1.75 - 3.86) | 3.03  (1.80 - 4.30) | 3.30  (1.88 - 4.95) | 3.47 (  1.91 - 5.48) |
| Expected offspring lost | 0.39  (0.11 - 0.79) | 1.10  (0.35 - 2.02) | 1.51  (0.53 - 2.56) | 1.96  (0.70 - 3.19) | 2.80  (1.12 - 4.26) | 3.57  (1.50 - 5.19) |

**Table S12.** Breakdown of datasets used in this study. Outcome variables are listed on the left and predictor variables (infection metrics; visible cysts, *Taenia* antigen detection in urine, and *Taenia* antigen detection in plasma) are listed on the top, with sample and individual numbers reflecting the sample size for each analysis using those intersecting datasets.

| ***T. serialis* metric** | | **cysts** | **urine Ag** | **plasma Ag** |
| --- | --- | --- | --- | --- |
| **tenure length** | # intervals (months) | 3,067 | | x |
|  | # individuals | 96 | | x |
|  | years | 2006 – 2023 | 2013-2020 | x |
| **fecal hormones** | # samples | 45 | 298 | 56 |
|  | # individuals | 39 | | |
|  | years | 2008 – 2020 | 2013-2020 | x |
| **plasma hormones** | # samples | x | x | 36 |
|  | # individuals | *x* | *x* | 36 |
|  | years | x | x | 2017 – 2020 |

**Table S13.** Plausible models included in AICc-based model selection for the faecal androgens analysis (K = number of parameters, AICc = corrected Akaike Information Criterion, Delta AICc = difference between model AICc and lowest AICc in the set, AICc Wt = relative likelihood of the model, Cum Wt = running total of AICcs, LL = model log likelihood). Final model is italicized.

| **Model** | **K** | **AICc** | **DeltaAICc** | **AICcWt** | **Cum.Wt** | **LL** |
| --- | --- | --- | --- | --- | --- | --- |
| log(T) ~ infection + status*poly(age) + season + 1\|ID + 1\|year | 15 | 528.36 | 0 | 0.35 | 0.35 | -248.55 |
| log(T) ~ infection + season + status + 1\|ID + 1\|year | 9 | 528.6 | 0.24 | 0.31 | 0.66 | -255.07 |
| *log(T) ~ infection* status + season + 1\|ID + 1\|year* | *11* | *529.5* | *1.14* | *0.2* | *0.86* | *-253.41* |
| log(T) ~ infection + season*status + poly(age) + 1\|ID + 1\|year | 15 | 532.13 | 3.77 | 0.05 | 0.92 | -250.44 |
| log(T) ~ infection + season + status + poly(age) + 1\|ID + 1\|year | 11 | 532.4 | 4.04 | 0.05 | 0.96 | -254.86 |
| log(T) ~ infection* status + poly(age) + season + 1\|ID + 1\|year | 13 | 532.9 | 4.54 | 0.04 | 1 | -252.98 |
| log(T) ~ infection + scale(Rain90) + status + 1\|ID + 1\|year | 8 | 548.11 | 19.75 | 0 | 1 | -265.87 |
| log(T) ~ infection + status*poly(age) + 1\|ID + 1\|year | 13 | 548.94 | 20.58 | 0 | 1 | -261 |
| log(T) ~ infection + status*poly(age) + scale(Rain90) + 1\|ID + 1\|year | 14 | 549.24 | 20.88 | 0 | 1 | -260.07 |
| log(T) ~ infection + scale(Rain90)*status + poly(age) + 1\|ID + 1\|year | 12 | 550.12 | 21.76 | 0 | 1 | -262.66 |
| log(T) ~ infection + status + poly(age) + 1\|ID + 1\|year | 9 | 550.97 | 22.61 | 0 | 1 | -266.25 |
| log(T) ~ infection + scale(Rain90) + status + poly(age) + 1\|ID + 1\|year | 10 | 551.98 | 23.62 | 0 | 1 | -265.71 |
| log(T) ~ infection* status + poly(age) + 1\|ID + 1\|year | 11 | 552.3 | 23.94 | 0 | 1 | -264.81 |
| log(T) ~ infection* status + poly(age) + scale(Rain90) + 1\|ID + 1\|year | 12 | 553.37 | 25.01 | 0 | 1 | -264.28 |
| log(T) ~ infection + season + poly(age) + 1\|ID + 1\|year | 9 | 553.56 | 25.2 | 0 | 1 | -267.55 |
| log(T) ~ infection + scale(Rain90) + poly(age) + 1\|ID + 1\|year | 8 | 567.63 | 39.27 | 0 | 1 | -275.63 |
